# Prognostic Value of Late Gadolinium Enhancement Cardiovascular Magnetic Resonance in Cardiac Amyloidosis

**DOI:** 10.1161/CIRCULATIONAHA.115.016567

**Published:** 2015-10-19

**Authors:** Marianna Fontana, Silvia Pica, Patricia Reant, Amna Abdel-Gadir, Thomas A. Treibel, Sanjay M. Banypersad, Viviana Maestrini, William Barcella, Stefania Rosmini, Heerajnarain Bulluck, Rabya H. Sayed, Ketna Patel, Shameem Mamhood, Chiara Bucciarelli-Ducci, Carol J. Whelan, Anna S. Herrey, Helen J. Lachmann, Ashutosh D. Wechalekar, Charlotte H. Manisty, Eric B. Schelbert, Peter Kellman, Julian D. Gillmore, Philip N. Hawkins, James C. Moon

**Affiliations:** From Heart Hospital, London, UK (M.F., S.P., P.R., A.A.-G., T.A.T., S.M.B., V.M., S.R., H.B., A.S.H., C.H.M., J.C.M.); Institute of Cardiovascular Science (M.F., A.A.-G., T.A.T., S.M.B., H.B., J.C.M.) and Department of Statistical Science (W.B.), University College London, UK; National Amyloidosis Centre, University College London, Royal Free Hospital, London, UK (M.F., S.M.B., R.H.S., K.P., S.M., C.J.W., H.J.L., A.D.W., J.D.G., P.N.H.); Bristol Heart Institute, University of Bristol, UK (C.B.-D.); University of Pittsburgh School of Medicine, PA (E.B.S.); and National Heart, Lung and Blood Institute, National Institutes of Health, Bethesda, MD (P.K.).

**Keywords:** amyloidosis, cardiac imaging techniques, magnetic resonance imaging, prognosis

## Abstract

The prognosis and treatment of the 2 main types of cardiac amyloidosis, immunoglobulin light chain (AL) and transthyretin (ATTR) amyloidosis, are substantially influenced by cardiac involvement. Cardiovascular magnetic resonance with late gadolinium enhancement (LGE) is a reference standard for the diagnosis of cardiac amyloidosis, but its potential for stratifying risk is unknown.

**Methods and Results—:**

Two hundred fifty prospectively recruited subjects, 122 patients with ATTR amyloid, 9 asymptomatic mutation carriers, and 119 patients with AL amyloidosis, underwent LGE cardiovascular magnetic resonance. Subjects were followed up for a mean of 24±13 months. LGE was performed with phase-sensitive inversion recovery (PSIR) and without (magnitude only). These were compared with extracellular volume measured with T1 mapping. PSIR was superior to magnitude-only inversion recovery LGE because PSIR always nulled the tissue (blood or myocardium) with the longest T1 (least gadolinium). LGE was classified into 3 patterns: none, subendocardial, and transmural, which were associated with increasing amyloid burden as defined by extracellular volume (*P*<0.0001), with transitions from none to subendocardial LGE at an extracellular volume of 0.40 to 0.43 (AL) and 0.39 to 0.40 (ATTR) and to transmural at 0.48 to 0.55 (AL) and 0.47 to 0.59 (ATTR). Sixty-seven patients (27%) died. Transmural LGE predicted death (hazard ratio, 5.4; 95% confidence interval, 2.1–13.7; *P*<0.0001) and remained independent after adjustment for N-terminal pro-brain natriuretic peptide, ejection fraction, stroke volume index, E/E′, and left ventricular mass index (hazard ratio, 4.1; 95% confidence interval, 1.3–13.1; *P*<0.05).

**Conclusions—:**

There is a continuum of cardiac involvement in systemic AL and ATTR amyloidosis. Transmural LGE is determined reliably by PSIR and represents advanced cardiac amyloidosis. The PSIR technique provides incremental information on outcome even after adjustment for known prognostic factors.

## Abstract 2

Supplemental Digital Content is available in the text.

The prognosis of immunoglobulin light-chain (AL or primary systemic) and transthyretin (ATTR) amyloidosis is substantially influenced by the presence and severity of cardiac involvement, which then governs therapeutic strategies.^1,2^ Although blood biomarkers are useful guides for risk stratification,^3^ they are not specific for cardiac involvement, and current strategies do not ascertain all patients at risk. Mortality, despite treatment progress, remains high.^4–7^ Over the last decade, new chemotherapy regimens and stem cell transplantation have been associated with improved survival in patients with AL amyloidosis, but the prognosis remains poor in those with cardiac involvement, which also contributes substantially to treatment-related morbidity and mortality. There remains a large unmet need for improved noninvasive criteria to stratify risk in selecting optimal therapy while avoiding serious toxicities.

Editorial see p 1525

Clinical Perspective on p 1579

Cardiac amyloid deposition represents a key process in amyloid pathophysiology.^8,9^ Cardiovascular magnetic resonance (CMR) with late gadolinium enhancement (LGE) identifies myocardial infiltration: after the administration of contrast, CMR shows a characteristic pattern of global subendocardial LGE coupled with abnormal myocardial and blood-pool gadolinium kinetics.^10,11^ However, despite excellent diagnostic accuracy for the presence of amyloid, conflicting results have been reported for the prognostic impact on AL amyloidosis, and no studies have been published in ATTR amyloidosis.^12–19^ Newer techniques, particularly phase-sensitive inversion recovery (PSIR), an LGE image reconstruction technique that is less sensitive to operator choice of null point and renders signal intensity truly T1 weighted, may better reflect extent of cardiac involvement^20^ and thereby improve risk stratification.

We report here a prospective CMR study conducted in amyloidosis in which we investigated the prognostic value of LGE in 250 consecutive CMR-eligible subjects. The aims of the study were to assess the LGE patterns and the benefit of new more robust approaches (PSIR), the correlation with the cardiac amyloid burden, and the prognostic impact of LGE in both AL and ATTR cardiac amyloidosis.

## Methods

### Amyloidosis Patients

Subjects were prospectively recruited at the National Amyloidosis Center, Royal Free Hospital, London, UK, from 2010 to 2014 (Figure I in the online-only Data Supplement). Outcome (dead/alive) was ascertained from death certificates. A total of 250 patients were categorized into 3 groups. The first group included 119 subjects with biopsy-proven systemic AL amyloid (77 male, 65%; age, 62±10 years), with biopsies from the myocardium (n=7, 6%) or other tissues (n=112, 94%). The second group comprised 122 consecutive, consenting patients with ATTR amyloidosis (101 male, 83%; age, 71±11years). Sixty-nine percent (n=84) had histological proof of ATTR amyloidosis by Congo red and immunohistochemical staining of myocardial (n=35, 29%) or other (n=49, 40%) tissues. The presence of cardiac amyloid was defined by the presence of ATTR amyloid in a myocardial biopsy or positive technetium-labeled bone scintigraphy using 3,3-diphosphono-1,2-propanodicarboxylicacid (DPD scintigraphy). All subjects underwent sequencing of exons 2, 3, and 4 of the *TTR* gene. The third group was made up of 9 subjects with amyloidogenic *TTR* gene mutations (3 male, 33%; age, 47±6 years) defined as individuals with no evidence of clinical disease (no cardiac uptake on DPD scintigraphy and normal echocardiography, CMR, N-terminal pro-brain natriuretic peptide [NT-proBNP], and troponin T).

### Exclusion Criteria

We excluded all patients with contraindications to CMR: glomerular filtration rate <30 mL/min and CMR-incompatible devices. All ethics were approved by the University College London/University College London Hospital Joint Committees on the Ethics of Human Research Committee, and all participants provided written informed consent.

### CMR Image Acquisition

All subjects underwent standard CMR on a 1.5-T clinical scanner (Avanto, Siemens Healthcare, Erlangen, Germany). Within a standard clinical scan (pilots, transverse white- and black-blood images, cines images to assess volumes and mass), LGE imaging was acquired with magnitude-only inversion recovery (MAG-IR) and PSIR sequence reconstructions in 43% of patients.

T1 measurement was performed with the use of the shortened modified look-locker inversion recovery sequence (ShMOLLI)^21^ with regions of interest drawn in the 4-chamber view at the level of the basal and mid inferoseptum (2 segments, large region of interest).^22^ After a bolus of gadoterate meglumine (0.1 mmol/kg, gadolinium-DOTA, Dotarem, Guerbet S.A. France) and standard LGE imaging (standard fast low-angle shot inversion recovery or balanced steady state free precession sequence with MAG-IR and PSIR reconstruction), the patient was removed from the scanner. The extracellular volume (ECV) measurement approach used equilibrium CMR with a primed infusion: At 15 minutes after bolus, an infusion at a rate of 0.0011 mmol·kg^−1^·min^−1^ contrast (equivalent to 0.1 mmol/kg over 90 minutes) was given. Between 45 and 80 minutes after bolus, the patient was returned to the scanner with the infusion continuing, and the T1 measurement was repeated using the same parameters of the precontrast ShMOLLI sequence.

### CMR LGE Interpretation

During interpretation, before our adoption of PSIR for all amyloidosis patients, because myocardial nulling can be difficult in the presence of amyloid, any confusion with MAG-IR images was resolved by selecting the images that most matched the postcontrast T1 maps, with “bright” LGE expected to correlate with areas with the lowest postcontrast T1 (ie, the highest gadolinium concentration, the highest interstitial expansion).

**Figure 1.**
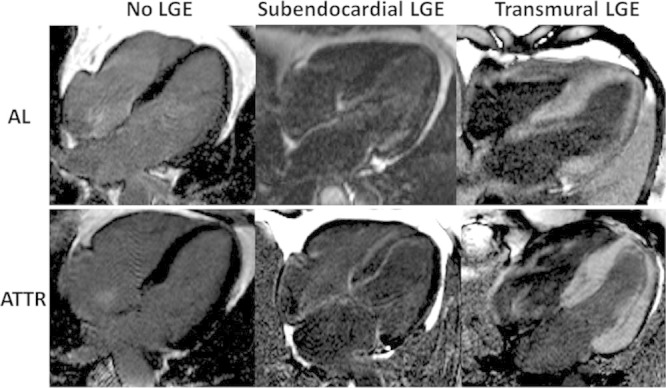
Characteristic phase-sensitive inversion recovery late gadolinium enhancement (LGE) patterns in 3 patients with immunoglobulin light-chain amyloidosis (AL) and 3 patients with transthyretin amyloidosis (ATTR). **Left**, No LGE; **middle**, subendocardial LGE; **right**, transmural LGE.

The LGE pattern was classified by 2 different observers (M.F. and S.P.) into 3 groups according to the degree of transmurality: group 1, no LGE; group 2, subendocardial LGE (when there was global subendocardial involvement but no transmural LGE); and group 3, transmural LGE (when the LGE was extending transmurally; Figure 1). Thus, a patient with basal transmural LGE but apical subendocardial LGE would be classified as transmural LGE.

### CMR PSIR Versus MAG-IR

**Figure 2.**
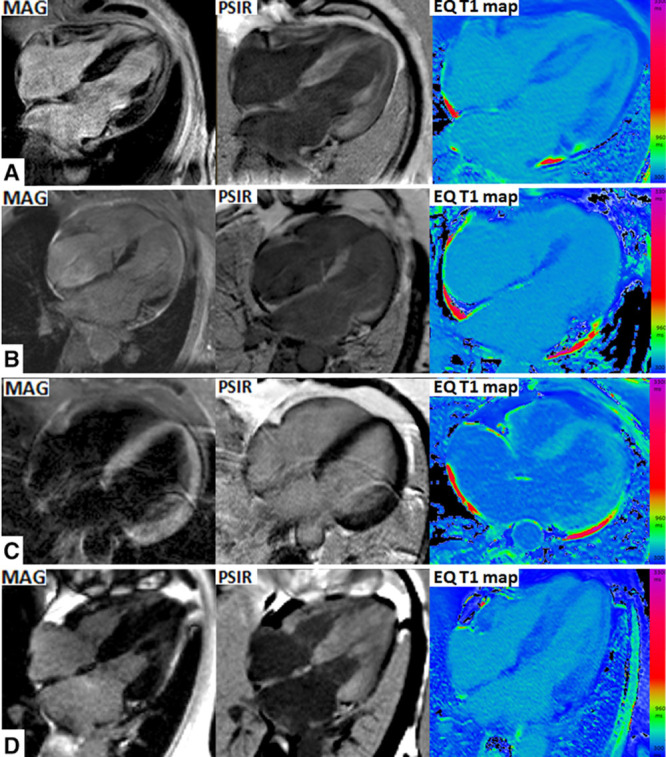
Characteristic cardiovascular magnetic resonance scans. Late gadolinium enhancement (LGE) with magnitude reconstruction (**left**); LGE with phase-sensitive inversion recovery reconstruction (PSIR; **middle**); and postcontrast shortened modified look-locker inversion recovery sequence (ShMOLLI) T1 maps (**right**). On PSIR, there is 100% concordance between myocardial T1 and LGE: first, areas of low T1 (darkest blue) and focal areas of LGE; second, where myocardial T1 is lower than blood T1, global LGE is demonstrated; and third, where myocardial T1 is higher than blood T1, no LGE is demonstrated. On magnitude-only inversion recovery (MAG) images, discordance is present in all 4 of these cases: mid myocardial rather than subendocardial (**A**), apical rather than basal (**B**), transmural LGE rather than normal (**C**), and normal rather than transmural (**D**).

A sample of 100 images (50 PSIR, 50 MAG-IR reconstruction) was analyzed for concordance or discordance with the postcontrast T1 maps that were used as the truth standard (Figure 2). We considered that nulled tissue should be the tissue with the least contrast (longest T1 on the postcontrast T1 map). This means that a normal subject should have nulled myocardium; a high-infiltration amyloid patient should have bright myocardium (transmural) and nulled blood, with the possibility of intermediate blood and myocardium nulling concurrently (typically with “bright” endocardium). This is discussed further in the figure legends and Discussion.

### Statistical Analysis

Statistical analysis was performed with IBM SPSS Statistics version 19 (IBM, Somers, NY) and the R programming language (http://www.r-project.org/). All continuous variables were normally distributed (Shapiro-Wilk) except for NT-proBNP and troponin T, which were therefore ln-transformed for bivariate testing. These are presented as mean±SD, and nontransformed NT-proBNP is presented as median and quartiles 1 through 3. Comparisons between groups were performed by 2-way ANOVA with post hoc Bonferroni correction. The χ^2^ test or Fisher exact test was used to compare discrete data as appropriate. Correlations between parameters were assessed with the Pearson *r* or Spearman ρ. To assess the agreement of the assignment of the LGE pattern by 2 different observers, the intraclass correlation coefficient was calculated. Statistical significance was defined as *P*<0.05.

Survival was evaluated with Cox proportional hazards regression analysis, providing estimated hazard ratios with 95% confidence intervals and Kaplan–Meier curves. Variables selected a priori for clinical relevance and first explored with univariate Cox regression were entered into the multivariable models. Multivariable models evaluated the independent predictive value of LGE above other clinically and statistically significant covariates. The Harrell C statistic was calculated for the different models.

## Results

### Study Population

The details of the 250 subjects are shown in Table 1. At the time of scanning, the AL amyloidosis cohort had 46 new untreated (to date) patients, 21 patients undergoing second- or third-line therapy, and 52 stable patients (complete or very good response, 80%; stable partial response, 20%). UK first-line therapy at the time of this study was typically cyclophosphamide, thalidomide, and dexamethasone or cyclophosphamide, bortezomib, and dexamethasone. Relapse therapy was typically cyclophosphamide, bortezomib, and dexamethasone or a lenalidomide-containing regimen. The *TTR* mutations were as follows: V122I, n=23; T60A, n=13; V30M, n=10; E54G, n=2; S77Y, n=2; E89K, n=2; and D38Y, G47V, E89K, I84S, I107F, and L12P, n=1 each. Of the 9 asymptomatic individuals with *TTR* mutations, 5 had *TTR* V30M, 3 had T60A, and 1 had S77Y.

**Table 1.**
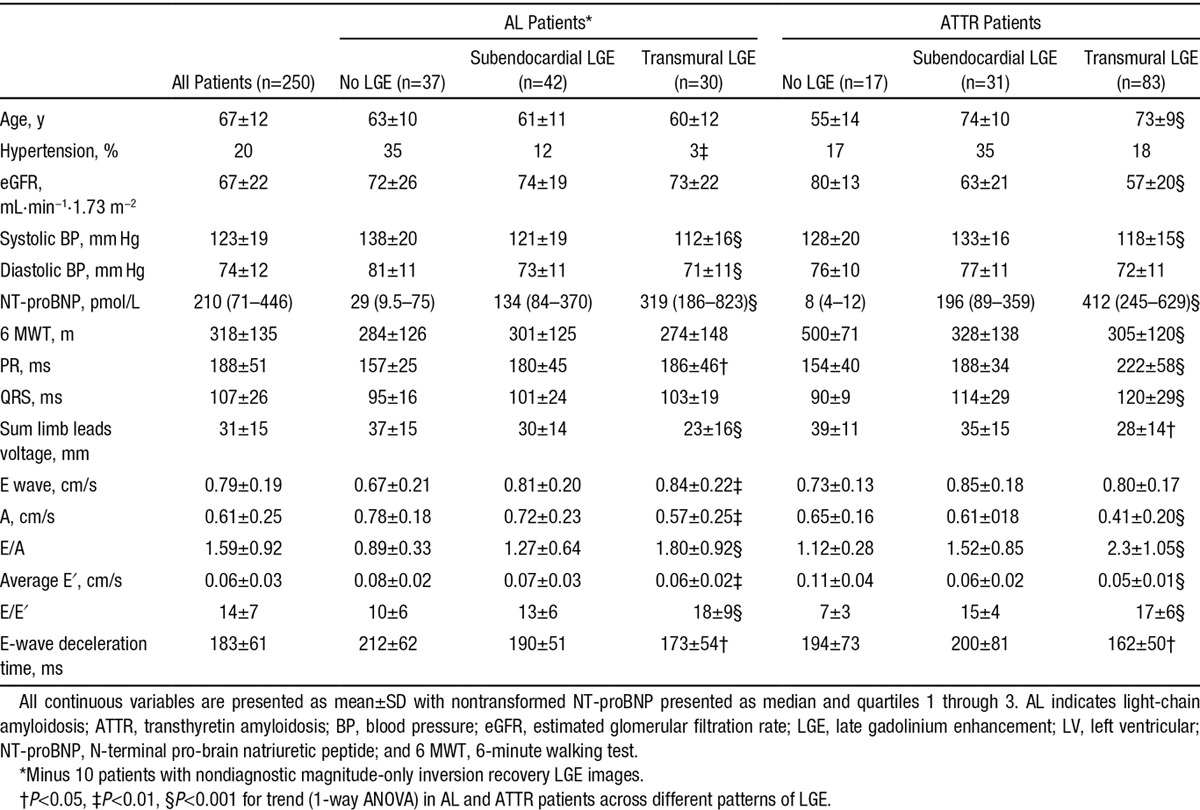
Main Clinical Characteristics and Echocardiographic and ECG Findings in Patients With AL and ATTR Amyloidosis According to the LGE Pattern

### MAG-IR Versus PSIR

**Figure 3.**
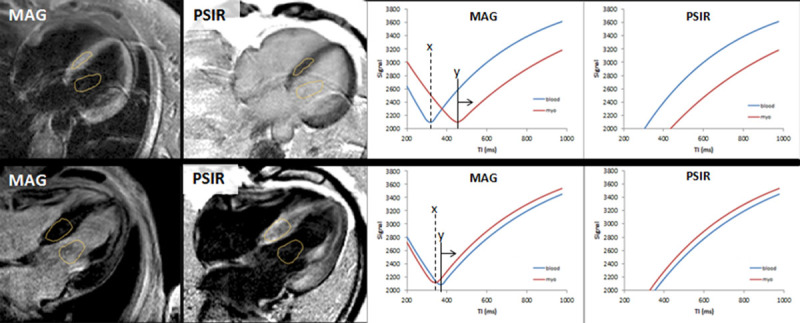
Two patients (**top** and **bottom**) with magnitude-only inversion recovery (MAG) and phase-sensitive inversion recovery reconstruction (PSIR) late gadolinium enhancement (LGE) reconstruction images (**left**). In both patients, the MAG and PSIR are discordant with opposite LGE patterns. Only one can be correct. The tissue to null is the one with the slowest T1 recovery (ie, the least gadolinium). **Right**, Signal intensity curves as the TI varies for MAG and PSIR. How the operator sets the TI matters in MAG imaging but not in PSIR. The operator set the TI for both patients at X, nulling the wrong tissue. The image would have been correct only if the operator had set the TI greater than Y. With PSIR, the TI could have been set anywhere, and the tissue with the least gadolinium has lower signal and will be nulled after windowing.

MAG-IR LGE and T1 maps were discordant in 57% (in which the operator was selecting the inversion time according to his/her best judgment), meaning that operator TI selection was mainly incorrect. Ten patients with MAG-IR only had no LGE images that matched the T1 mapping for classification (implying that the operator systematically kept the TI incorrect for the whole scan). All patients with PSIR LGE had diagnostic images. PSIR LGE and T1 maps were never (0%) discordant (Figures 2 and 3). MAG-IR could be incorrect in 3 ways: inappropriately nulling global LGE (Figure 2D), particularly the highest ECV cases; getting the incorrect distribution (especially making LGE apical rather than basal; Figure 2A and 2B); or creating transmural LGE where there should be global nulling (and the ECV was low; Figure 2C). With PSIR, the longest T1 tissue after windowing is always nulled.

### LGE Pattern and Correlation With ECV

**Figure 4.**
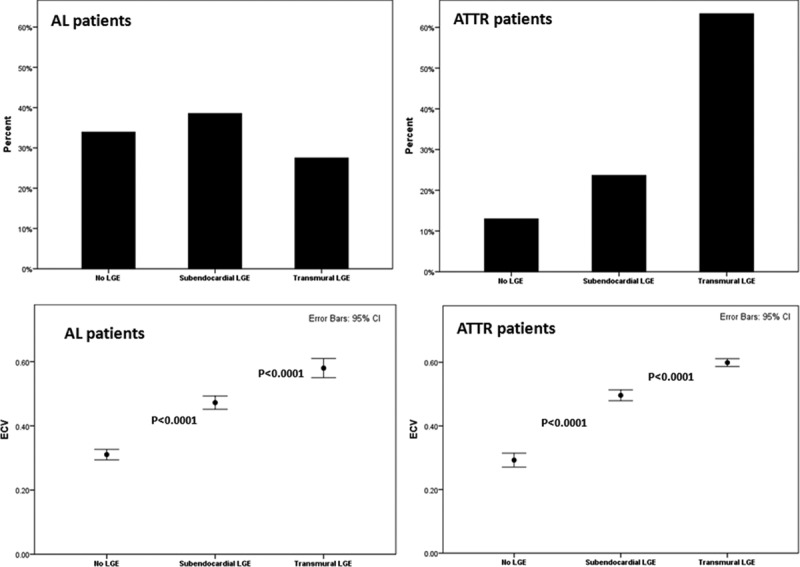
Late gadolinium enhancement (LGE) pattern correlation with amyloid burden. **Top**, Histograms showing the prevalence of the different LGE patterns in patients with immunoglobulin light-chain amyloidosis (AL) and patients with transthyretin amyloidosis (ATTR). **Bottom**, Correlation with the amyloid burden measured as extracellular volume (ECV) in AL and ATTR patients. Bonferroni adjustment was applied. CI indicates confidence interval.

Three patterns of LGE are observed: no LGE, subendocardial LGE, and transmural LGE (Figure 1). There was good agreement in the assignment of these patterns between 2 observers (intraclass correlation coefficient, 0.97; 95% confidence interval, 0.97–098). All patterns were present in AL and ATTR cardiac amyloidosis (Figure 1) but to different extents, with subendocardial LGE being more prevalent in AL (39% in AL versus 24% in ATTR; *P*<0.05) and transmural LGE more prevalent in ATTR (27% in AL versus 63%; *P*<0.0001; Figure 4).

Increasing LGE (none, subendocardial, transmural) was associated with increasing ECV (AL: 0.31±0.04, 0.47±0.06, and 0.58±0.07; ATTR: 0.29±0.04, 0.50±0.05, and 0.60±0.05; *P*<0.0001 for both; Figure 4). In ATTR, this correlated also with DPD grade (*P*<0.0001). Apparent transitions are evident, with subendocardial LGE appearing at an ECV of 0.40 to 0.43 for AL and 0.39 to 0.40 for ATTR and transmural at an ECV of 0.48 to 0.55 for AL and 0.47 to 0.59 for ATTR. However, 39% of the patients with no LGE had ECV elevation compared with normal range (ECV elevation between 0.32 and 0.40). Of the patients with no LGE and increased ECV, 4 patients had mutant ATTR (and DPD was grade 1 in 3 patients and grade 0 in 1 patient), and 17 patients had AL amyloidosis.

Increasing LGE (none, subendocardial, transmural) was associated in both AL and ATTR with lower systolic blood pressure, ECG changes (prolonged PR interval, prolonged QRS in ATTR), increased NT-proBNP, structural and functional changes (increased LV mass, increased end-systolic volume, decreased stroke volume, decreased ejection fraction, left atrial dilatation), increasingly abnormal tissue characterization (elevated native T1 and ECV; Table 2), and more severe echocardiographic diastolic dysfunction. In ATTR, increasing LGE was also associated with decreased functional capacity (6-minute walking test).

**Table 2.**
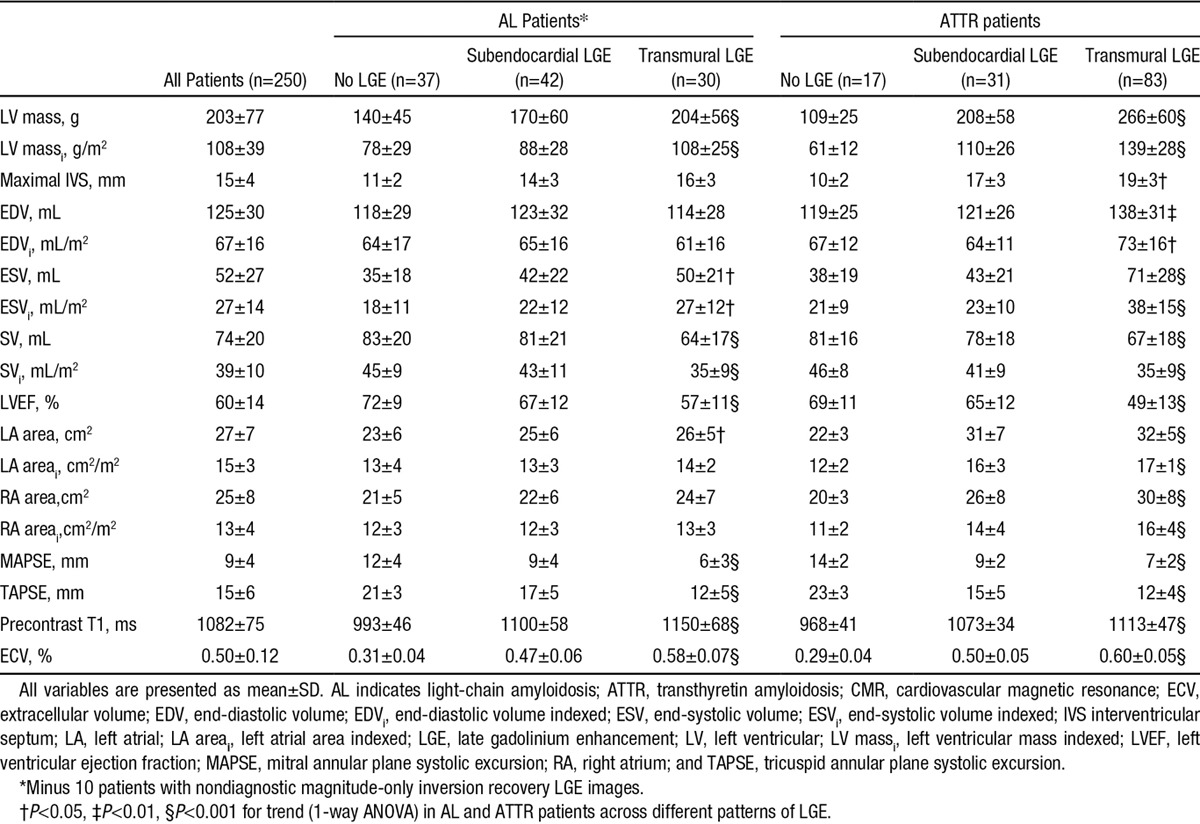
CMR Findings in Patients With AL and ATTR Amyloidosis According to the LGE Pattern

### LGE Pattern and Prognosis

**Figure 5.**
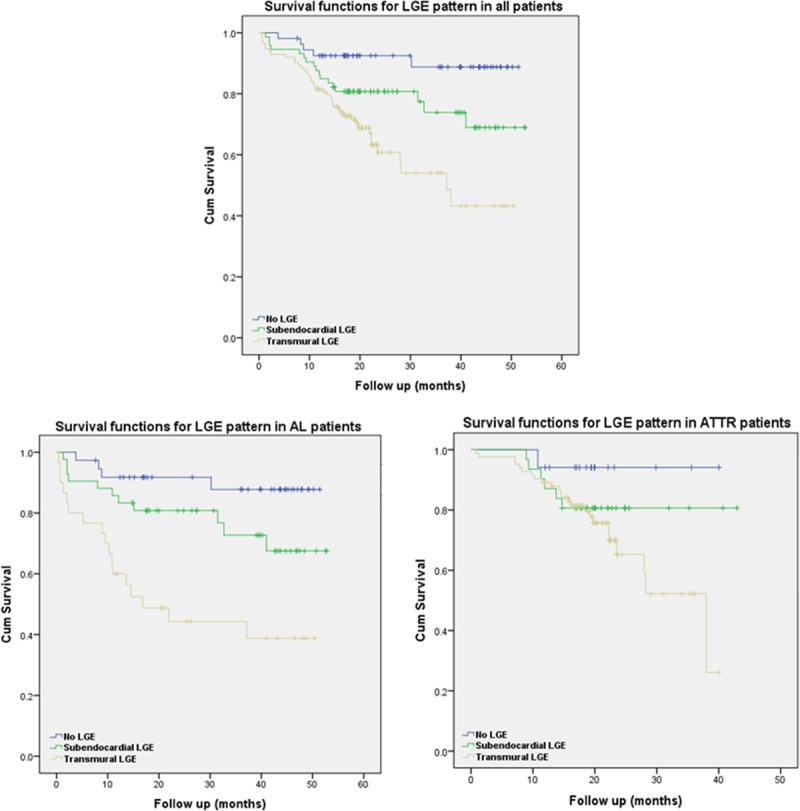
Kaplan–Meier curves for late gadolinium enhancement (LGE) patterns in all patients (**top**), patients with immunoglobulin light-chain amyloidosis (AL; **bottom left**), and patients with transthyretin amyloidosis (ATTR; **bottom right**).

At follow-up (mean, 24±13 months), 67 of 250 patients (27%) had died. Transmural LGE was a significant predictor of mortality in the overall population (hazard ratio, 5.4; 95% confidence interval, 2.1–13.7; *P*<0.0001; Figure 5).

The survival curves indicates that there is an ≈92% chance of survival at 24 months in patients with a no LGE (92% in AL, 94% in ATTR) compared with 81% for patients with subendocardial LGE (81% in AL, 81% in ATTR) and 61% with transmural LGE (45% in AL, 65% in ATTR). The median survival in patients with transmural LGE was 17 months in AL and 38 months in ATTR. Transmural LGE was significantly associated with mortality (hazard ratio, 4.1; 95% confidence interval, 1.3–13.1; *P*<0.05) in multivariable Cox models that included NT-proBNP, ejection fraction, stroke volume indexed, E/E′, and left ventricular mass indexed (Troponin results were not available in all patients). NT-ProBNP and stroke volume indexed also remained independently predictive (Table 3). Harrell C statistics for this model was 0.72.

**Table 3.**
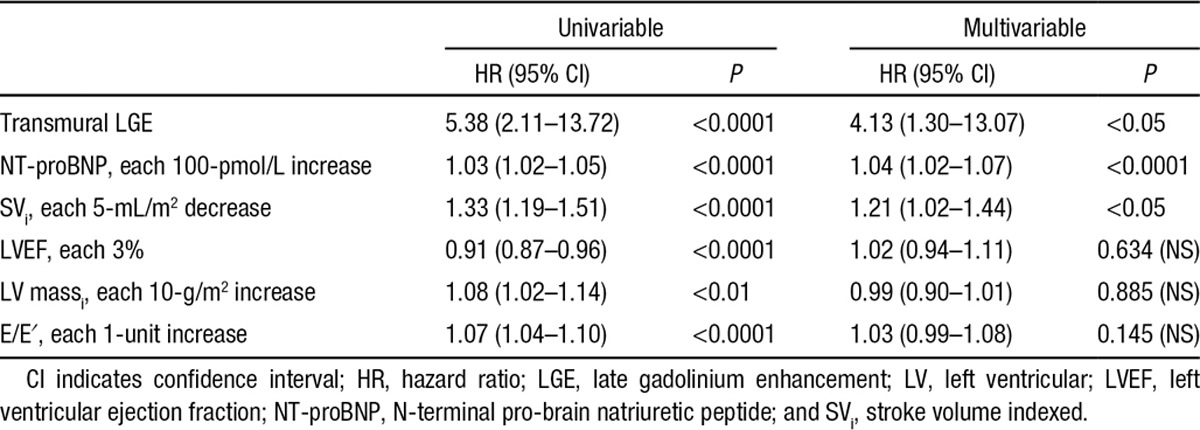
Univariate and Multivariate Analyses of Risk of Death in the Overall Population

The Harrell C statistics of a comparable pre-CMR model including demographics, systolic and diastolic function parameters, and biomarkers (age, ejection fraction, E/E′, NT-ProBNP, interventricular septal wall thickness) was 0.67.

## Discussion

Cardiac infiltration is the chief driver of prognosis in systemic amyloidosis, and stratification of patients is essential for prognosis and optimal management, including selection of patients to receive aggressive higher-risk therapies and to minimize cardiac toxicities. Echocardiography, once the gold standard cardiac investigation in amyloidosis, has limited sensitivity and specificity, and risk stratification currently places great emphasis on blood biomarkers. However, these strategies do not identify all amyloidosis patients at risk, and the findings of studies evaluating cardiac involvement by CMR have been conflicting.^4–7,19^ Recently, considerable interest has emerged in using LGE to improve the risk stratification model,^12–19^ but studies in AL cardiac amyloidosis have been few, mostly small, and retrospective; have used nonstandardized LGE approaches; and have produced inconsistent results.^10,12,13,15,16,19^ No studies have been published in ATTR amyloidosis.

In the present study, the largest CMR study in amyloidosis to date, we showed that misleading results using the MAG-IR LGE technique were likely to account for the conflicting finding that have previously been published. By convention, areas with the most contrast should be displayed as bright on LGE imaging (ie, shortest T1 on T1 maps). For amyloidosis, myocardium can contain more gadolinium than blood. Under those circumstances, myocardium should appear globally bright (transmural LGE). It is a property of MAG imaging that the signal is highly dependent and nonlinear with a user-defined choice of the TI (time to null; Figure 2), and images can be “inverted” with the wrong TI choice. When all of the myocardium is abnormal (seen frequently in cardiac amyloidosis), the abnormal myocardium could be wrongly nulled because the MAG-IR relies on “nulling” what is perceived to be normal myocardium. This limitation was quantified in our study by comparison with a true standard of postcontrast T1. More important, this problem does not occur with the PSIR approach. PSIR substantially removes the issue of operator-selected inversion time and completely removes the potential for a “mirror image.” On a PSIR reconstruction when windowed by the operator, the tissue with the longest T1 (least gadolinium, ie, blood or myocardium) after windowing is always nulled. The practical results include the following: first, if myocardium is globally nulled by PSIR and the ECV is less than blood and <0.4 to 0.43, any amyloid present (detectable by an ECV >0.32) is not extensive. Second, above this value, LGE areas appear particularly in the subendocardium. PSIR LGE areas are the areas of most amyloid deposition in the heart. Third, above an ECV of 0.47 to 0.59, when blood has less gadolinium than myocardium and blood is nulled (myocardium appears uniformly bright), heterogeneity is present but is swamped by all the myocardium becoming bright. Examples of MAG-IR errors are shown in Figure 2: mid myocardial rather than subendocardial (Figure 2A), apical rather than basal (Figure 2B), transmural LGE rather than normal (Figure 2C), and normal rather than transmural (Figure 2D).^20,23^ Accordingly, we believe that PSIR should be universally adopted for amyloid LGE imaging, particularly because PSIR-LGE is easily available from all scanner manufacturers (whereas T1 mapping is not yet).

**Figure 6.**
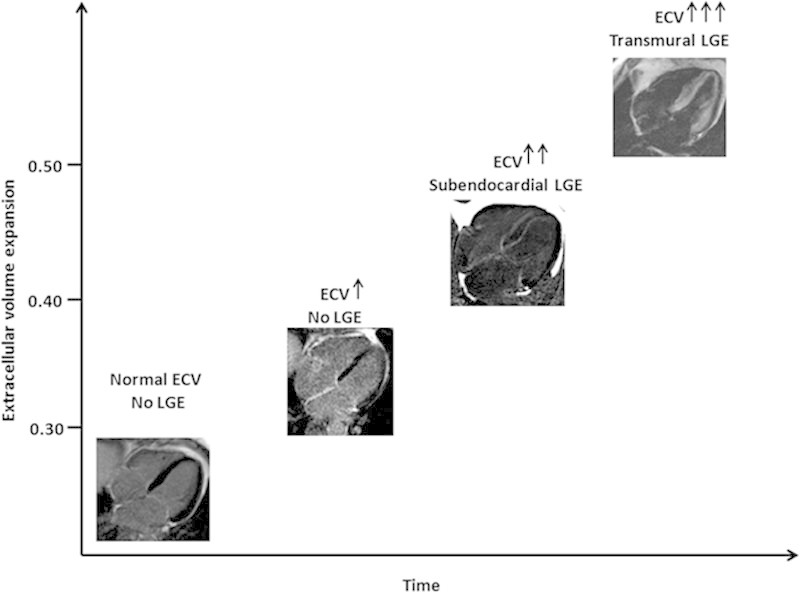
Hypothesized cardiac amyloid progression across time. When amyloid starts to accumulate, 3 steps can be identified: (1) no evidence of late gadolinium enhancement (LGE) but an increase in native T1 and extracellular volume (ECV), (2) a further increase in T1 and ECV and the appearance of subendocardial LGE; and (3) a further increase in native T1 and ECV and progression to transmural LGE.

With PSIR, 3 patterns were relatively easy to determine, and the frequently described LGE pattern of patchy LGE was not evident with PSIR (many of these on PSIR appeared to have transmural LGE). A key insight is that amyloid cardiac involvement is not dichotomous but a continuum from no LGE to subendocardial to transmural tracking increasing ECV (Figure 6).^24,25^ Transmural LGE appears to be the pattern that carries the most adverse prognosis. It is an important marker of all-cause mortality after adjustment for other relevant disease variables and regardless of treatment status (indeed regardless of whether patients are presenting at diagnosis or years into the disease process). Indeed, in ATTR, the majority of the deaths are in patients with transmural LGE (no LGE, subendocardial LGE, and transmural TGE: 1, 6, and 24 deaths, respectively).

Within this spectrum, the degree of involvement is important, with transmural LGE defining the high-risk group. The prevalence of patients in the different stages of disease progression (Figure 6) is different in AL and ATTR. Thirty-nine percent of the patients with no LGE had ECVs in the range of 0.32 to 0.4, particularly patients with AL amyloidosis. It is expected that ATTR patients must pass through this phase, but it is not clinically recognized. Early cardiac involvement in AL is detected through cardiac screening of AL patients presenting with extracardiac disease. The majority of ATTR patients (wild-type ATTR and mutant ATTR associated with the variant V122I) present with heart failure symptoms that appear only when advanced (LGE, mostly transmural, is invariably present). This has potential treatment implications. Currently, patients with subendocardial LGE may be classified as having cardiac involvement and denied therapies that are known to improve long-term survival but are contraindicated in cardiac involvement such as stem cell transplantations (AL), some chemotherapy regimens (AL), and liver transplantations (ATTR). More data are needed, and consideration of cardiac involvement as a continuum should provide insights into the impact of different degrees of cardiac infiltration, possibly resulting in changes in the current therapeutic approach. Within the transmural pattern, the median survival is significantly different in the 2 amyloid types: 17 months for AL and 38 months for ATTR. These findings support the concept that cardiac amyloid is not a disease of solely infiltration but may have superimposed toxicity (AL more than ATTR) or that the rate of accumulation is myotoxic, a contributor to different prognoses or AL and ATTR despite ATTR having higher degrees of left ventricular hypertrophy, cardiac dysfunction, and amyloid burden.^26^ T1 mapping techniques provide new insights into this, being able to follow the disease at 3 different levels, that is, infiltration (amyloid burden, ECV), edema (native T1), and myocyte response (intracellular volume), and providing new prognostic markers.^27^ These new biomarkers may aid diagnosis and risk stratifications and act as surrogate end points in clinical trials. However, the current limited availability and the technical challenges related to sequence- and vendor-specific differences limit the role of T1 mapping in routine clinical practice. The common use of the LGE technique in all clinical CMR scans and the availability of PSIR reconstruction on all different vendor platforms make LGE a robust and reliable approach for routine risk stratification of patients with cardiac amyloidosis.

Limitations of the study are that patients were at different treatment stages, with treatment reflecting current UK practice. Cardiac biopsy was present in only a minority of patients, but this cohort of patients was fully characterized with all other clinical investigative techniques currently available, including DPD scanning. This composite diagnostic pathway is known to provide high diagnostic accuracy. The causes of death are not known because patients die locally and the National Amyloidosis center receives only notification of death, not the cause of death. Although this study highlights the prognostic role of transmural LGE for risk stratification of patients with cardiac amyloidosis, further studies are needed to assess the direct correlation between patterns of LGE and treatment-related mortality.

## Sources of Funding

Dr Fontana is supported by a Clinical Research Training Fellowships from the British Heart Foundation (FS/12/56/29723). A. Abdel-Gadir is supported by the Rosetrees Trust. T.A. Treibel is supported by the National Institute for Health Research (DRF-2013-06-102). Dr Moon is supported by the Higher Education Funding Council for England. Dr Bucciarelli-Ducci is supported by the Bristol NIHR Biomedical Research Unit. This work was undertaken at the University College London Hospital and University College London, which received a proportion of funding from the Department of Health’s National Institute for Health Research Biomedical Research Centres funding scheme.

## Disclosures

None.

## Supplementary Material

## CLINICAL PERSPECTIVE

Cardiac infiltration is the chief driver of prognosis in systemic amyloidosis. Its assessment aids in the selection of patients to receive (or not) aggressive chemotherapy, stem cell therapy, and newer therapies. Currently, stratification uses blood biomarkers and echocardiography, but echocardiography has limited sensitivity and specificity, particularly in apparently early disease and when confounders (such as hypertension) are present. Cardiovascular magnetic resonance shows promise with the late gadolinium enhancement (LGE) technique to visualize infiltration, but the technique has always been difficult in cardiac amyloidosis because of difficult nulling, with both early and advanced disease being potentially misclassified. Here, using T1 mapping as a truth standard (bright myocardium should have the most contrast present), we show in 250 patients with both immunoglobulin light chain and transthyretin amyloidosis that a new but widely available LGE technique, phase-sensitive inversion recovery, completely solves the nulling problems in amyloidosis. With the use of phase-sensitive inversion recovery, the longest T1 tissue after windowing is always nulled. Three LGE patterns are present in cardiac amyloidosis: no LGE, subendocardial LGE, and transmural TGE. The transmural LGE pattern is associated with the highest infiltration and carries the most adverse prognosis. It is a marker of all-cause mortality, even after adjustment for other relevant disease variables. These results suggest that cardiac infiltration is a continuum with the degree of involvement being measurable and important, with transmural LGE defining the high-risk group and the subendocardial LGE group being a potential key group to focus on for therapy.
