# Supplementary material for: Prognostic Value of Late Gadolinium Enhancement Cardiovascular Magnetic Resonance in Cardiac Amyloidosis
Source: Circulation. 2015 Oct 19;132(16):1570–9. doi: 10.1161/CIRCULATIONAHA.115.016567 (PMC4606985; doi:10.1161/CIRCULATIONAHA.115.016567)
Supplement: Supplementary file 1 [file cir-132-1570-s001.docx]

**SHORT COMMENTARY**

Cardiac infiltration is the chief driver of prognosis in systemic amyloidosis. Its assessment aids patient selection to receive (or not) aggressive chemotherapy, stem cell therapy and newer therapies. Currently, stratification uses blood biomarkers and echocardiography - but echocardiography has limited sensitivity and specificity particularly in apparently early disease and where confounders are present (such as hypertension). Cardiovascular Magnetic Resonance shows promise with the Late Gadolinium Enhancement (LGE) technique to visualise infiltration, but the technique has always been difficult in cardiac amyloidosis due to difficult nulling, with both early and advanced disease being potentially mis-classified. Here we show in 250 patients with both AL and TTR amyloidosis, using T1 mapping as a truth standard (bright myocardium should have the most contrast present), that a new but widely available LGE technique Phase Sensitive Inversion Recovery (PSIR) completely solves the nulling problems in amyloidosis. Using PSIR, the longest T1 tissue after windowing is always nulled. Three LGE patterns are present in cardiac amyloidosis– no LGE, subendocardial and transmural. Transmural LGE is the pattern that is associated with the highest infiltration and carries the most adverse prognosis – it is a marker of all cause mortality, even after adjustment for other relevant disease variables. The results suggest that cardiac infiltration is a continuum with the degree of involvement being measurable and important - with transmural LGE defining the high risk group and the subendocardial LGE group being a potential key group to focus on for therapy.
