# Supplementary figures and images for: Prognostic Value of Late Gadolinium Enhancement Cardiovascular Magnetic Resonance in Cardiac Amyloidosis
Source: Circulation. 2015 Oct 19;132(16):1570–9. doi: 10.1161/CIRCULATIONAHA.115.016567 (PMC4606985; doi:10.1161/CIRCULATIONAHA.115.016567)

## SUPPLEMENTAL MATERIAL

**Supplemental Figure 1.** Consort diagram.

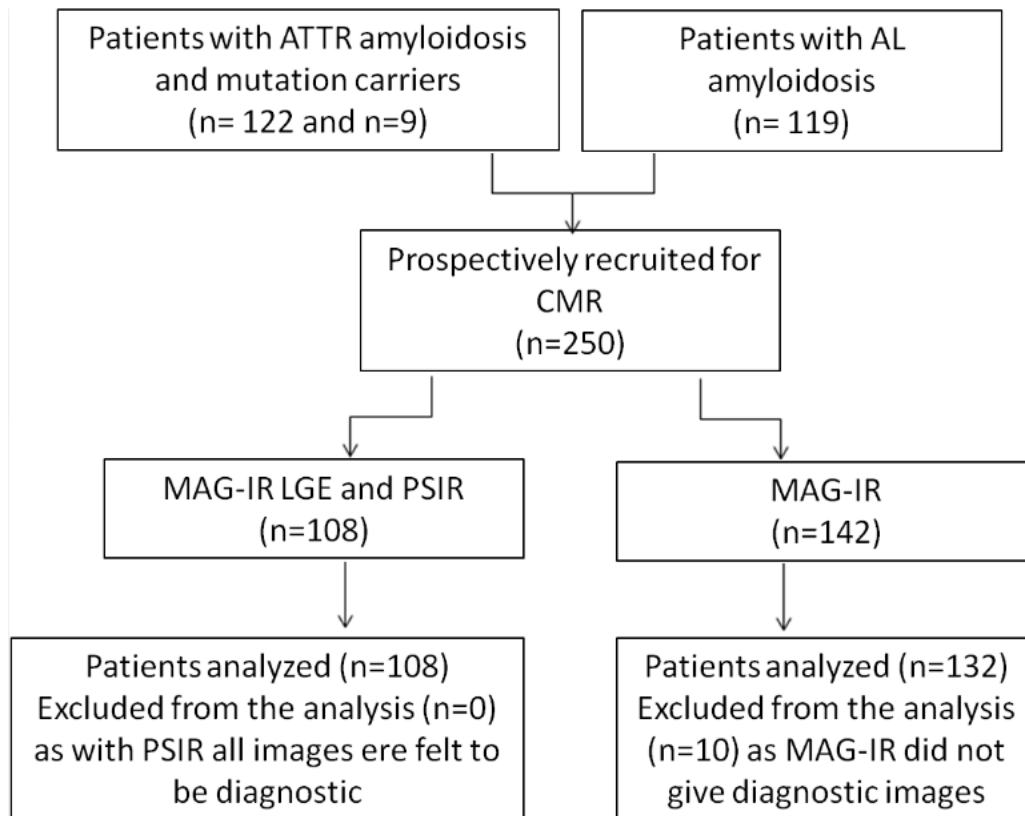

Supplement: Supplementary file 2 [file cir-132-1570-s002.pdf]
